# Aspiration and methylprednisolone injection to the cavity with IV cannula needle in the treatment of volar wrist ganglia: New technique

**DOI:** 10.12669/pjms.291.2655

**Published:** 2013

**Authors:** Murat Korkmaz, Hayati Ozturk, Dilsad Amanvermez Senarslan, Yalcin Erdogan

**Affiliations:** 1 Murat Korkmaz, Assistant Professor, Dept. of Orthopedics and Traumatology, Bozok University Medical Faculty, Yozgat, Turkey; 2 Hayati Ozturk, Associate Professor, Dept. of Orthopedics and Traumatology, Cumhuriyet University Medical Faculty, Sivas, Turkey.; 3 Dilsad Amanvermez Senarslan, Lecturer, Department of Cardiovascular Surgery, Bozok University Medical Faculty, Yozgat, Turkey; 4 Yalcin Erdogan, Assistant Professor, Department of Family Physician, Bozok University Medical Faculty, Yozgat, Turkey

**Keywords:** Wrist ganglia, Volar, Aspiration, Corticosteroid injection, Radial artery

## Abstract

***Objective:*** There are several types of treatment modalities for wrist ganglions. The aim of the study was to assess the effectiveness of cyst aspiration and methyl prednisolone acetate injection with double IV cannula rather than sharp pointed needle, as a new technique in the treatment of volar ganglia.

***Methodology:*** The study involves total of 19 patients who received treatment by aspiration and methyl prednisolone injection into the cavity. Two IV cannulas are pricked to the cystic cavity. Cyst fluid is drained by the distally placed IV cannula meanwhile injecting methyl prednisolone by proximally placed IV cannula. The patient records and follow-up reports are retrospectively investigated. The patient age, sex, site of the cyst, the treatment that was applied, adjacency to the artery and the nerves and recurrence are recorded. Mean follow up time was 2.1±0.5 years.

***Results:*** The study involved 19 patients that received aspiration treatment for volar ganglion cysts between January 2004 and December 2009. There were 12 (63.2%) female and 7 (36.8%) male subject with volar wrist ganglion cyst. The mean age of patients was 27.63±6.6 years. Fourteen (73.7%) patients of total had cysts close to the artery. We didn’t observe any complication related to methyl prednisolone injection and arterial ischemia. Recurrence was observed in three (15.8%) patients.

***Conclusion:*** This method has lower recurrence rate than other aspiration therapy with sharp pointed needle. We prefer to use IV cannula needle for cyst aspiration and steroid injection in treatment of volar ganglia before any surgical intervention.

## Introduction

 Ganglion is a common cystic lesion that is benign, fluid-filled capsule which can be seen all over the body.^1^ The ganglia originate from the joint capsule, tendon, tendon sheath and rarely from the artery wall.^2^^,^^3^ Wrist ganglia cysts usually develop in the consequence of fluid leak out where placed within the sheath that surrounds the wrist tendons. It becomes a cystic structure that contains identical fluid with the normal fluid found within a joint or a tendon sheath. Wrist ganglia are most commonly observed in the dorsal aspect of the hand and less often in the volar aspect of the hand.^2^ The cyst may communicate with the joint by a pedicle.

 Ganglia are more common in women than in men. They are typically seen in between the third and the sixth decades of life.^4^ Average size ranges from 1 to 3 cm diameter but there are case reports stating larger sizes of ganglia. The patients are usually asymptomatic however the pain may be present when the cyst applies pressure on surrounding tissues, especially on a nerve. Sometimes volar ganglia may cause paresthesia arising from compression of ulnar or radial nerves or their branches. The lump is generally smooth, fairly tense and fixed. In the treatment of the ganglia there are three ways; 1) Conservative therapy which is convenient for small sized, asympthomatic lumps. 2) Aspiration 3) Surgical excision. Aspiration is generally performed via single or double sharp pointed needle. Firstly, cyst fluid is aspirated and then steroid is injected into cyst cavity. The recurrence rate of this technique is relatively high as between 59%- 68% for dorsal ganglion (even with recurrence rate of 88% for volar ganglion).^5^^,^^6^

 The purpose of the study was to assess the effectiveness of cyst aspiration and methyl prednisolone acetate injection with double IV cannula rather than sharp pointed needle, as a new technique in the treatment of volar ganglia.

## Methodology

 The study involved 19 patients that received aspiration and methyl prednisolone acetate injection treatment for volar ganglion cysts between January 2004 and December 2009. The patient records and follow-up reports were investigated. The patient age, gender, cyst localization, adjacency to the artery and the nerves, recurrences and complications were recorded. Patients were followed by physical examination and ultrasonography for the period of two years after the treatment of aspiration. Cysts were examined for diameter, tenseness and characteristic of the cyst fluid via physical examination and ultrasound screening. Two IV cannulas were pricked to the cystic cavity; one of the IV cannula size of 20 Gauge (G) tip was pricked distal part of the cyst for evacuation of the cavity. At the proximal part of the cyst the other 22 G IV cannula was pricked to inject methyl prednisolone into the cavity. Of two IV cannulas, the metal parts were retracted, only plastic parts remained in the cystic cavity. Meanwhile evacuating the cavity by IV cannula from the distal part, methyl prednisolone (Depo-Medrol®, 40 mg methyl prednisolone) was injected via the IV cannula at the proximal part. This process was maintained until all cystic fluid was drained and white colored methyl prednisolone was seen in the needle that placed distally.

**Fig.1:**
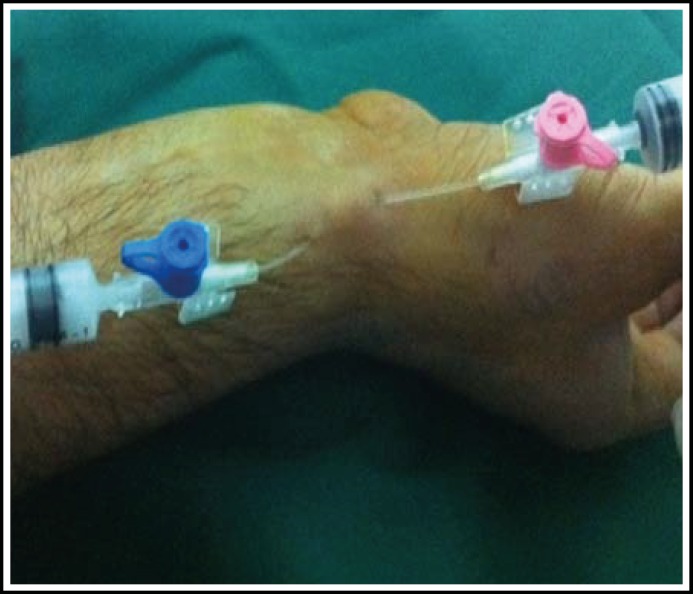
Two IV cannulas are pricked to the cystic cavity. The cavity evacuated by the distally placed IV cannula & methyl prednisolone is injected by the proximal IV cannula

## Results

 Patients mean follow up time was 2.1±0.5 years. There were 12 (63.2%) female and 7 (36.8%) male subject with volar wrist ganglia. The mean age of the patients was 27.63±6.6 years. All the cysts were smaller from 2.5 centimeter (cm) diameter. Of ganglia 11 (57.9%) were in right hand and 8 (42.1%) were in left hand. The cysts close to the radial artery were detected in 14 (73.7%) patients by Doppler ultrasonography. No complication related to methyl prednisolone acetate injection was observed. Recurrences were noted in 3 (15.8%) patients (Table-I). There was no difference between male and female in terms of recurrence (Fisher’s exact test, p=0.7). The relapsed ganlion cysts were treated by surgical excision.

**Table-I:** Demographic and clinical data

Male / Female (n) (%)	7 (36.8)/12(63.2)
Age, mean±S.D. (years)	27.63±6.64
Mean follow-up time, mean±S.D. (years)	2.1±0.5
Recurrence (n) (%)	3 (15.8)
Side of wrist Right/left (n) (%)	11(57.9)/8(42.1)
Adjacency arteries and nerves +/- (n)(%)	14(73.7)/ 5(26.3)

S.D: Standard deviation

## Discussion

 Ganglia are the most common cause of a palpable mass in the wrist and hand.^7^ Treatment options include various procedures; observation, aspiration, aspiration with sclerotherapy, arthroscopic resection and surgical excision. The pain, limitation of activity, nerve and arterial injury and higher recurrence rate are encountered in result of aggressive treatments.^8^ In the present study, we achieved favorable results with cyst aspiration and injection of methyl prednisolone acetate treatment with not sharp pointed but IV cannula needle in patients with volar wrist ganglia. In follow-up period of this new aspiration technique with plastic IV cannula needle, no complication, low recurrence rate and decreased need for surgical intervention were observed. 

 Various treatments with different complication rates are reported in the literature. Faithfull et al showed that in 28% of patients who had surgical intervention were unsatisfied due to persistent pain, limitation of function.^9^ Gundes et al. found the mean complication rate was 56% for volar ganglia and 12.5% for dorsal ganglia. They stated palmar cutaneous branch of the median nerve in two patients and the palmar superficial branch of the radial artery laceration in two patients.^10^ In another study, injury to the median palmar cutaneous nerve in 10%, injury to the radial artery in 5% and wrist stiffness in 12.5% of the patients are declared.^11^ In a similar study parallel results were obtained.^12^ Gumus et al. showed that the procedure of sclerotherapy damages the lining of the main ganglion and causes severe fibrosis around the cyst.^13^ Furthermore thumb and index finger ischemia after aspiration and sclerosing agent injection in volar wrist ganglion has been reported. Palmar circulation did not recover and the digits became gangrenous with clear demarcation. The patients left thumb the distal phalanx had to be amputated.^14^ Another animal study explains the inherent dangers of this therapy with radiological and electron microscopic data.^15^ No nerve-arterial damage or limitation of movement in wrist was determined in this aspiration technique with plastic IV cannula needle.

 The recurrence rates of surgical and arthroscopic interventions for volar ganglion treatment are reported between 14-28% in the literature.^9^^,^^16^^,^^17^ In the previous studies, recurrence rates for simple aspiration and aspiration plus steroid injection therapies are 59-68% and 40% respectively.^5^^,^^6^^,^^18^ In the present study we determined the recurrence rate of 15.8% for the new technique of cyst aspiration and steroid injection with plastic IV cannula needle. This favorable recurrence rate of this method is lower than the one of other method using sharp pointed needle. Even this low recurrence rate in our study is almost same with the recurrence rates of surgical and arthroscopic interventions.

 Arthroscopic resections are reported recently by orthopedic surgeons. After the operation there was no impairment of wrist motion, function and neurovascular complication.^19^^,^^20^ Recent studies suggest arthroscopic resection as an effective and safe method with less postoperative morbidity and better cosmetic results for dorsal ganglia. But they also emphasize that volar ganglia should still be treated by open operation because arthroscopic resection is difficult technically.^21^^,^^22^ The use of IV cannulas enables direct entrance into the cystic cavity. After observing the cyst fluid coming by the IV cannula, sharp-pointed needle part is retracted and plastic part is less harmful for artery and nerves surrounding the cyst. Therefore complications related to vicinal structures are less encountered. The reason of our low recurrence rate is total aspiration of the cyst fluid until the white colored methyl prednisolone acetate was seen in syringe and usage of cortisone.

 In conclusion, we have evaluated the method of aspiration and injection of methyl prednisolone acetate into the cavity by IV cannula needle as a new technique. This method has lower recurrence rate than other aspiration therapy with sharp pointed needle. We prefer to use IV cannula needle for cyst aspiration and steroid injection in treatment of volar ganglia before any surgical intervention. Because it is simple, noninvasive, safe and effective method.
